# Supplementary material for: Confidence bands and hypothesis tests for hit enrichment curves
Source: J Cheminform. 2022 Jul 28;14:50. doi: 10.1186/s13321-022-00629-0 (PMC9334420; doi:10.1186/s13321-022-00629-0)
Supplement: Supplementary file 1 — Additional file 1. Proof of Theorem 1 [file 13321_2022_629_MOESM1_ESM.pdf]

SUPPLEMENTARY MATERIAL

Appendix: Proof of Theorem 1

Let $\mathbf{W}_i = (X_i, S_{1i}, S_{2i})$, where $\mathbf{W}_1, ..., \mathbf{W}_n$ is a random sample from a probability distribution P on a measurable space $(\mathcal{W}, \mathcal{A})$ for $\mathcal{W} = \{0,1\} \times \mathbb{R} \times \mathbb{R}$. To arrive at the convergence in distribution result for $\widehat{\theta}_{1r} - \widehat{\theta}_{2r}$, we need to establish Hadamard differentiability of $\widehat{\theta}_{1r} - \widehat{\theta}_{2r}$. To do so, we use the fact that under Conditions 1 and 2, both $\widehat{\theta}_{1r}$ and $\widehat{\theta}_{2r}$ are Hadamard differentiable (Jiang & Zhao 2015). Hadamard differentiability of $\widehat{\theta}_{1r} - \widehat{\theta}_{2r}$ is implied by the definition of Hadamard differentiability (3.9.1, van der Vaart & Wellner (1996)).

The influence function associated with $\widehat{\theta}_{jr}$ is $L_j(\boldsymbol{W}) = H_j$, where $H_j = (X - \Lambda_{jr})(A_{jr} - \theta_{jr})/\pi_+$ and $A_{jr} = \mathrm{I}(S_j > t_{jr})$, for $j = 1, 2$. Applying the chain rule for influence functions, we obtain the influence function for $\widehat{\theta}_{1r} - \widehat{\theta}_{2r}$ as $L(\boldsymbol{W}) = H_1 - H_2$. Consequently,

$$\sqrt{n}\Big\{(\widehat{\theta}_{1r} - \widehat{\theta}_{2r}) - (\theta_{1r} - \theta_{2r})\Big\} \xrightarrow{d} N(0, Var(H_1 - H_2))$$

as $n \to \infty$. The required variance expression is obtained as $Var(H_1 - H_2) = Var(H_1) + Var(H_2) - 2Cov(H_1, H_2)$. Jiang & Zhao (2015) already obtained the expression for $Var(H_j)$ as

$$\frac{\theta_{jr}(1-\theta_{jr})}{\pi_+}\Big[1 - 2\Lambda_{jr} + \frac{\Lambda_{jr}^2(1-r)r}{\pi_+\theta_{jr}(1-\theta_{jr})}\Big].$$

We now obtain $Cov(H_1, H_2)$. Using the law of total covariance, we have that:

$$\begin{aligned}
&Cov\big(\left(X - \Lambda_{1r}\right)\left(A_{1r} - \theta_{1r}\right), \left(X - \Lambda_{2r}\right)\left(A_{2r} - \theta_{2r}\right)\big) \\
&= E\big[Cov\big(\left(X - \Lambda_{1r}\right)\left(A_{1r} - \theta_{1r}\right), \left(X - \Lambda_{2r}\right)\left(A_{2r} - \theta_{2r}\right) \mid X\big)\big] \\
&\quad + Cov\big(E\left[\left(X - \Lambda_{1r}\right)\left(A_{1r} - \theta_{1r}\right) \mid X\right], E\left[\left(X - \Lambda_{2r}\right)\left(A_{2r} - \theta_{2r}\right) \mid X\right]\big).
\end{aligned}$$

For the first term we have that:

$$
\begin{aligned}
&E\big[Cov\big((X-\Lambda_{1r})(A_{1r}-\theta_{1r}), (X-\Lambda_{2r})(A_{2r}-\theta_{2r}) \mid X\big)\big] \\
&= \pi_+ Cov\big((X-\Lambda_{1r})(A_{1r}-\theta_{1r}), (X-\Lambda_{2r})(A_{2r}-\theta_{2r}) \mid X=1\big) \\
&\quad + (1-\pi_+)\, Cov\big((X-\Lambda_{1r})(A_{1r}-\theta_{1r}), (X-\Lambda_{2r})(A_{2r}-\theta_{2r}) \mid X=0\big) \\
&= \pi_+ (1-\Lambda_{1r})(1-\Lambda_{2r})\, Cov(A_{1r}, A_{2r}|X=1) + (1-\pi_+)(\Lambda_{1r})(\Lambda_{2r})\, Cov(A_{1r}, A_{2r}|X=0) \\
&= \pi_+ (1-\Lambda_{1r})(1-\Lambda_{2r})(\theta_{12\cdot r} - \theta_{1r}\theta_{2r}) + (1-\pi_+)(\Lambda_{1r})(\Lambda_{2r})(\theta'_{12\cdot r} - \theta'_{1r}\theta'_{2r}),
\end{aligned}
$$

where $\theta'_{jr} = P(S_j > t_{jr}|-)$ for $j \in \{1,2\}$ and $\theta'_{12\cdot r} = P(S_1 > t_{1r}, S_2 > t_{2r}|-)$. For the second term we have:

$$
\begin{aligned}
&Cov\big(E[(X-\Lambda_{1r})(A_{1r}-\theta_{1r}) \mid X], E[(X-\Lambda_{2r})(A_{2r}-\theta_{2r}) \mid X]\big) \\
&= E\big(E[(X-\Lambda_{1r})(A_{1r}-\theta_{1r}) \mid X] \cdot E[(X-\Lambda_{2r})(A_{2r}-\theta_{2r}) \mid X]\big) \\
&\quad - (E[E[(X-\Lambda_{1r})(A_{1r}-\theta_{1r}) \mid X]]) \cdot (E[E[(X-\Lambda_{2r})(A_{2r}-\theta_{2r}) \mid X]]) \\
&= (1-\pi_+) \cdot E[(X-\Lambda_{1r})(A_{1r}-\theta_{1r}) \mid X=0] \cdot E[(X-\Lambda_{2r})(A_{2r}-\theta_{2r}) \mid X=0] \\
&\quad - (1-\pi_+)\, E[(X-\Lambda_{1r})(A_{1r}-\theta_{1r}) \mid X=0] \cdot (1-\pi_+)\, E[(X-\Lambda_{2r})(A_{2r}-\theta_{2r}) \mid X=0] \\
&= (\pi_+)(1-\pi_+)\Lambda_{1r}\Lambda_{2r}(\theta'_{1r}-\theta_{1r})(\theta'_{2r}-\theta_{2r}).
\end{aligned}
$$

Thus,

$$
\begin{aligned}
Cov(H_1, H_2) = \frac{1}{\pi_+^2}\Bigg\{ & \pi_+ (1-\Lambda_{1r})(1-\Lambda_{2r})(\theta_{12\cdot r} - \theta_{1r}\theta_{2r}) + (1-\pi_+)(\Lambda_{1r})(\Lambda_{2r})(\theta'_{12\cdot r} - \theta'_{1r}\theta'_{2r}) \\
& + \pi_+(1-\pi_+)\Lambda_{1r}\Lambda_{2r}(\theta'_{1r}-\theta_{1r})(\theta'_{2r}-\theta_{2r}) \Bigg\}.
\end{aligned}
$$

The expression in brackets can be expanded as a bivariate polynomial function of Λ_{1r} and Λ_{2r}:

$$\pi_+(1-\Lambda_{1r}-\Lambda_{2r})(\theta_{12\cdot r}-\theta_{1r}\theta_{2r})$$
$$+(\Lambda_{1r}\Lambda_{2r})\Bigg\{\pi_+(\theta_{12\cdot r}-\theta_{1r}\theta_{2r})+(1-\pi_+)(\theta'_{12\cdot r}-\theta'_{1r}\theta'_{2r})$$
$$+\pi_+(1-\pi_+)(\theta'_{1r}-\theta_{1r})(\theta'_{2r}-\theta_{2r})\Bigg\}.$$

The coefficient of $\Lambda_{1r}\Lambda_{2r}$ is complicated but noticing that:

$$E[Cov(A_{1r},A_{2r}|X)]=\pi_+(\theta_{12\cdot r}-\theta_{1r}\theta_{2r})+(1-\pi_+)(\theta'_{12\cdot r}-\theta'_{1r}\theta'_{2r})$$

and that:

$$\begin{aligned}&Cov\left(E\left[A_{1r}|X\right],E\left[A_{2r}|X\right]\right)\\&=E\left[E\left[A_{1r}|X\right]\cdot E\left[A_{2r}|X\right]\right]-E\left[E\left[A_{1r}|X\right]\right]\cdot E\left[E\left[A_{2r}|X\right]\right]\\&=\pi_+\theta_{1r}\theta_{2r}+(1-\pi_+)\,\theta'_{1r}\theta'_{2r}-\left(\pi_+\theta_{1r}+(1-\pi_+)\,\theta'_{1r}\right)\left(\pi_+\theta_{2r}+(1-\pi_+)\,\theta'_{2r}\right)\\&=\pi_+\left(1-\pi_+\right)\left(\theta'_{1r}-\theta_{1r}\right)\left(\theta'_{2r}-\theta_{2r}\right)\end{aligned}$$

implies that the coefficient of $\Lambda_{1r}\Lambda_{2r}$ is $E\left[Cov\left(A_{1r},A_{2r}|X\right)\right]+Cov\left(E\left[A_{1r}|X\right],E\left[A_{2r}|X\right]\right)=Cov(A_{1r},A_{2r})=\gamma_{12\cdot r}-r^2$.

Thus:

$$\begin{aligned}&Cov\left(H_1,H_2\right)\\&=\frac{1}{\pi_+^2}\left(\pi_+(\theta_{12\cdot r}-\theta_{1r}\theta_{2r})(1-\Lambda_{1r}-\Lambda_{2r})+\left(\gamma_{12\cdot r}-r^2\right)\Lambda_{1r}\Lambda_{2r}\right)\\&=\frac{(\theta_{12\cdot r}-\theta_{1r}\theta_{2r})}{\pi_+}\left\{(1-\Lambda_{1r}-\Lambda_{2r})+\frac{\left(\gamma_{12\cdot r}-r^2\right)\Lambda_{1r}\Lambda_{2r}}{\pi_+\left(\theta_{12\cdot r}-\theta_{1r}\theta_{2r}\right)}\right\}.\end{aligned}$$

References

Jiang, W. & Zhao, Y. (2015), 'On asymptotic distributions and confidence intervals for lift measures in data mining', *Journal of the American Statistical Association* **110**(512), 1717–1725.

van der Vaart, A. W. & Wellner, J. A. (1996), *Weak Convergence and Empirical Processes*, Springer Series in Statistics, Springer New York, New York, NY.
